# Peripheral blood cells RNA-seq identifies differentially expressed gene network linked to lymphocyte subsets alterations and active lupus nephritis associated with declines in renal function

**DOI:** 10.1016/j.heliyon.2024.e32303

**Published:** 2024-06-02

**Authors:** Yi-Chen Chen, Hsin-Hui Yu, Ya-Chiao Hu, Yao-Hsu Yang, Yu-Tsan Lin, Li-Chieh Wang, Bor-Luen Chiang, Jyh-Hong Lee

**Affiliations:** a Fu Jen Catholic University Hospital, New Taipei City, Taiwan, China; b Department of Pediatrics, National Taiwan University Hospital and National Taiwan University College of Medicine, Taipei, Taiwan, China; c Graduate Institute of Clinical Medicine, National Taiwan University College of Medicine, Taipei, Taiwan, China

**Keywords:** Differentially expressed gene, Glomerular filtration rate, Lupus nephritis, Lymphocyte subset, RNA-seq, T lymphocyte

## Abstract

**Background:**

The aim of this study was to investigate whether quantitative changes in lymphocyte subsets and gene expression in peripheral blood (PB) cells are related to the clinical manifestations and pathogenesis of lupus nephritis (LN).

**Methods:**

We enrolled 95 pediatric-onset SLE patients with renal involvement who presented with 450 clinical episodes suspicious for LN flare. Percentages of lymphocyte subsets at each episode were determined. We stratified 55 of 95 patients as high or low subset group according to the median percentage of each lymphocyte subset and the association with changes in the eGFR (ΔeGFR) were analyzed. Peripheral blood bulk RNA-seq to identify differentially expressed genes (DEGs) in 9 active LN vs. 9 inactive LN patients and the DEG-derived network was constructed by Ingenuity Pathway Analysis (IPA).

**Results:**

The mean ΔeGFR of low NK-low memory CD4^+^ T-high naive CD4^+^ T group (31.01 mL/min/1.73 m^2^) was significantly greater than that of high NK-high memory CD4^+^ T-low naive CD4^+^ T group (11.83 mL/min/1.73 m^2^; *P* = 0.0175). Kaplan-Meier analysis showed that the median time for ΔeGFR decline to mean ΔeGFR is approximately 10 years for high NK-high memory CD4^+^ T-low naive CD4^+^ T group and approximately 5 years for low NK-low memory CD4^+^ T-high naive CD4^+^ T group (log-rank test *P* = 0.0294).

**Conclusions:**

Our study highlighted important connections between DEG-derived network, lymphocyte subset composition, and disease status of LN and GN. A novel scoring system based on lymphocyte subset proportions effectively stratified patients into groups with differential risks for declining renal function.

## 1 Introduction

Systemic lupus erythematosus (SLE) is a clinically heterogeneous disease with varying clinical presentations and complex transcriptional signatures [1]. Factors such as genetic predisposition, proinflammatory and anti-inflammatory cytokines, defects in the complement system, autoantibodies, and lymphocyte subset abnormalities are known to contribute to the development of SLE. Studies have shown a close relationship between the production of autoantibodies and the abnormalities in lymphocyte subpopulations [2]. These abnormalities lead to the development and progression of SLE by promoting hypergammaglobulinemia and autoantibody production [3].

Despite significant advances in treating most autoimmune diseases over the past two decades, progress in SLE and lupus nephritis (LN) has lagged. Furthermore, numerous novel drugs have been tested for LN; however, none have shown efficacy in randomized controlled trials [4]. Moreover, intensive efforts have been made to identify biomarkers in the serum and urine of patients with LN; however, none of the identified markers are robust enough to replace renal biopsy. High-throughput technologies such as RNA sequencing (RNA-seq) offer ideal tools to delineate the mechanisms underlying this heterogeneous response [5]. The resulting data are expected to provide insights into distinct cell subpopulations as potential therapeutic targets for SLE [6].

Changes in peripheral blood (PB) lymphocyte subsets have been reported in patients with SLE; however, the data are often conflicting, including data on increased, decreased, or normal CD4^+^ T cell counts [7,8]. Studies have reported an increase or a decrease in the ratio of CD4^+^/CD8^+^T cells in patients with SLE. A low CD4/CD8 ratio is a characteristic of LN [9], a frequent complication of SLE and a predictor of SLE morbidity and mortality [4]. Additionally, a significant correlation has been reported between the decrease in natural killer (NK) cell count and the onset of both active renal affection and impairment of renal function [10,11]. The ratio and number of CD4^+^ lymphocytes are lower in patients with class IV LN than those in controls [12].

Arazi et al. identified subsets of leukocytes, including multiple populations of T, NK, and B cells, which demonstrated both pro- and anti-inflammatory activities in patients with LN. The study revealed a strong correlation between the expression signature of the genes in cells isolated from urine and that of the genes in immune cells in the blood [13]. The current understanding of the dysregulated molecular pathways in SLE mainly comes from unbiased analyses of blood cells, but it remains unclear to what extent the blood cell profile reflects inflamed tissue, such as the kidney in LN.

We hypothesized that outcomes in LN are associated with specific lymphocyte subsets in patients with SLE. To test this hypothesis, we aimed to investigate whether quantitative changes in lymphocyte subpopulations and their gene expression patterns are related to the clinical manifestations and pathophysiology of LN.

## 2 Results

### 2.1 Comparison of lymphocyte subsets in patients with different status of LN

Our study included 95 participants (Table 1), 45 of whom were ≥18 years old (41 females and 4 males) and 50 were <18 years old (43 females and 7 males). The mean duration of follow-up was approximately 2.49 years (2.49 ± 2.46 years). On average, each patient experienced approximately 2.5 episodes of inactive LN and 2.69 episodes of active LN during the follow-up period. Patients ≥18 years old had a lower estimated glomerular filtration rate (eGFR) than patients <18 years old. The main long-term treatments for the patients with SLE were Plaquenil and prednisolone, with MMF used as maintenance drugs.

**Table 1:** Demographic and laboratory characteristics of the systemic lupus erythematosus patients.

	all patients	≥18 years-old	<18 years-old
Case number	95	45	50
F:M	84:11	41:4	43:7
Age (years)	18.37 ± 7.437	24.93 ± 5.325	12.76 ± 3.106
WBC (K/μL)	6.371 ± 3.031 (0.08–22.02)	6.676 ± 2.815 (0.08–15.98)	6.146 ± 3.167 (0.45–22.02)
Hb (g/dL)	11.11 ± 1.982 (5.2–17.9)	11.12 ± 1.755 (6.8–17.9)	11.11 ± 2.138 (5.2–17.8)
Platelet (K/μL)	265.7 ± 117.2 (9–699)	265.1 ± 101.9 (14–522)	266.1 ± 127.6 (9–699)
ESR (mm/hr)	28.43 ± 24.51 (1–140)	28.95 ± 22.51 (2–127)	28.01 ± 26.02 (1–140)
CRP (mg/dL)	0.3968 ± 0.9157 (0.01–9.18)	0.4123 ± 1.004 (0.01–9.18)	0.3845 ± 0.8414 (0.02–6.02)
serum Creatinine (mg/dL)	0.9931 ± 1.143 (0.01–12.1)	1.094 ± 1.207 (0.3–9.5)	0.9126 ± 1.084 (0.01–12.1)
eGFR (mL/min/1.73 m^2^)	130.0 ± 66.48 (4.4–347.0)	107.8 ± 46.28 (8.6–221)	148.0 ± 74 (4.4–347.0)
SLEDAI	8.508 ± 5.308 (2–22)	7.843 ± 4.646 (2–21)	8.960 ± 5.679 (2–22)
anti-dsDNA (IU/mL)	458.5 ± 341.5 (17.93–1589)	429.9 ± 309.6 (30.37–1249)	477.8 ± 360.8 (17.93–1589)
C3 (mg/dL)	73.70 ± 26.91 (5.55–176)	75.50 ± 25.08 (25.8–176)	72.33 ± 28.2 (5.55–153.6)
C4 (mg/dL)	14.58 ± 9.805 (1.68–79.3)	13.74 ± 6.822 (2.44–43.1)	15.23 ± 11.57 (1.68–79.3)
Drugs (Prescription frequency, %)			
Plaquenil (mg)	213.7 ± 80.49 (87.56 %)	229.4 ± 70.57 (35.55 %)	203.0 ± 85.11 (52.00 %)
Prednisolone (mg)	17.29 ± 35.31 (81.78 %)	12.08 ± 20.6 (32.89 %)	20.79 ± 42.11 (48.89 %)
Mycophenolate Sodium (mg)	624.2 ± 349.1 (60.67 %)	604.8 ± 356.2 (24.89 %)	637.7 ± 344.6 (35.78 %)
Cyclosporine (mg)	42.71 ± 13.96 (18.89 %)	32.69 ± 11.77 (5.78 %)	47.12 ± 12.57 (13.11 %)
Azathioprine (mg)	48.68 ± 34.74 (5.56 %)	72.92 ± 34.47 (2.67 %)	26.31 ± 13.95 (2.89 %)

**Fig. 1:**
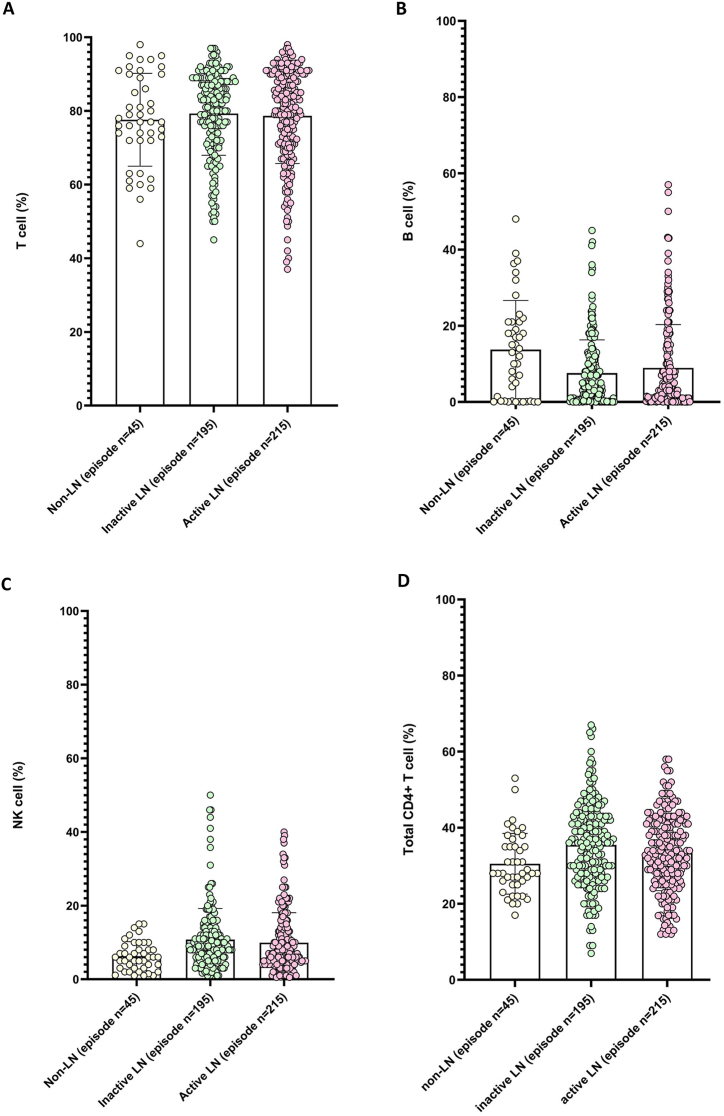

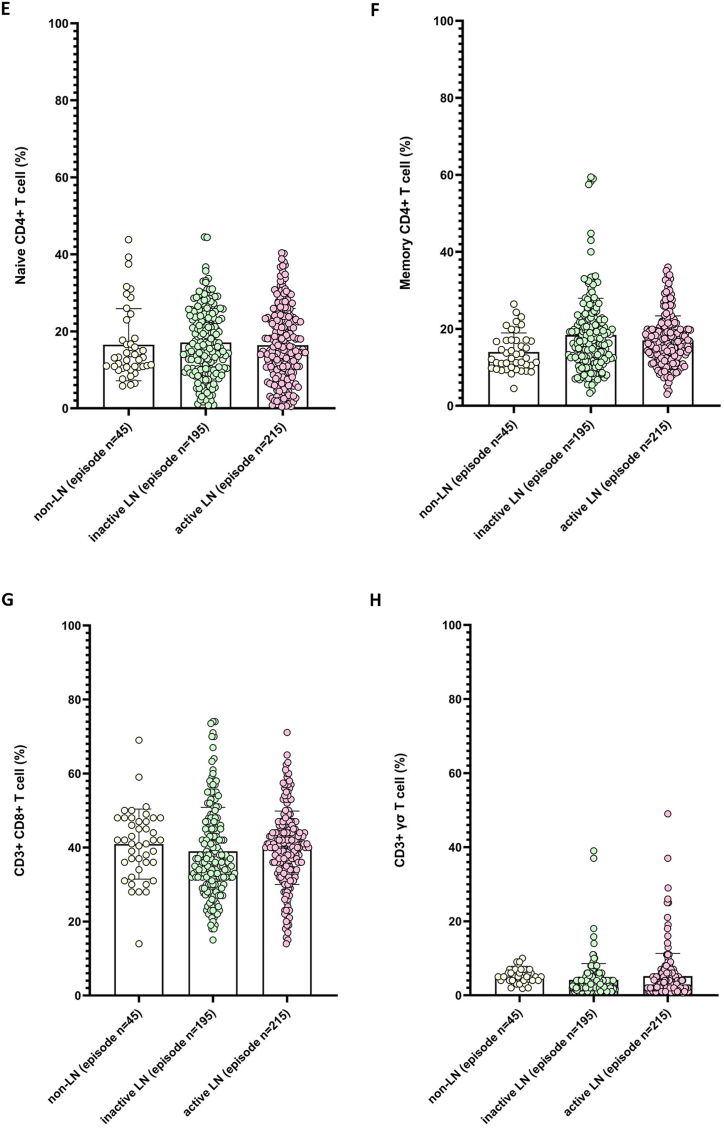
Comparison of Lymphocyte Subsets in Systemic Lupus Erythematosus (SLE) Patients across Different Lupus Nephritis (LN) Episodes. The data in this figure was based on 455 clinical episodes where lupus nephritis (LN) was suspected. Of these episodes, 45 were non-LN, 195 were inactive LN, and 215 were active LN. The percentages of the lymphocyte subset cells were compared between the three groups: (**A**) T lymphocytes, (**B**) B lymphocytes, (**C**) natural killer (NK) cells, (**D**) total CD4^+^ T lymphocytes, (**E**) naive CD4^+^ T lymphocytes, (**F**) memory CD4^+^ T lymphocytes, (**G**) CD3^+^CD8^+^ T lymphocytes, and (**H**) CD3^+^ γδ T lymphocytes. Horizontal solid lines represent means and ranges of ±SD.

To investigate the composition of the lymphocyte subsets, we compared the percentage of lymphocyte subsets among the non-LN, inactive-LN, and active-LN episode groups (Fig. 1A–H; Supplement Fig. 1). Patients with LN episodes showed higher percentages of NK, total CD4^+^ T, and memory CD4^+^ T cells than those without LN episodes. Patients with active LN episodes had lower percentages of NK and memory CD4^+^ T cells than those with inactive LN episodes. However, patients with active LN episodes had a higher percentage of B, CD3^+^ CD8^+^ T, and CD3^+^ γδ T cells than those with inactive LN episodes (Table 2). As previously reported, patients with LN have a lower frequency of CD3^+^CD4^+^ T cells and a higher percentage of CD3^+^CD8^+^ T cells (Fig. 1D and G; Table 1) [[14], [15], [16]]. Patients with active LN episodes had a higher Systemic Lupus Erythematosus Disease Activity Index (SLEDAI) score than those with inactive LN episodes (12.62 ± 4.849 vs. 5.072 ± 2.003, *P* < 0.0001), indicating disease activity was significantly associated with LN (Table 2).

**Table 2:** Comparative analysis of laboratory characteristics across different episodes in SLE patients with and without lupus nephritis (LN).

	Non-LN (episode n = 45)	Inactive LN (episode n = 195)	Active LN (episode n = 215)
**T cell (%)**	77.62 ± 12.61 (44–98 %)	79.31 ± 11.34 (45–97 %)	78.67 ± 12.95 (37–98 %)
**B cell (%)**	13.74 ± 12.87 (0–48 %)	7.573 ± 8.724 (0–45 %)	8.935 ± 11.39 (0–57 %)
**NK cell (%)**	6.293 ± 3.93 (1–15 %)	10.79 ± 8.426 (1–50 %)	9.920 ± 8.172 (0.5–40 %)
**Total CD4^+^** **T cell (%)**	30.49 ± 7.978 % (17–53 %)	35.48 ± 11.25 (6.96–67 %)	33.35 ± 9.92 (12–58 %)
**Naive CD4^+^** **T cell (%)**	16.51 ± 9.354 (5.8–43.8 %)	17.09 ± 8.862 (0.8–44.5 %)	16.42 ± 9.511 (0.4–40.4 %)
**Memory** **CD4^+^ T cell (%)**	13.98 ± 4.942 (4.5–26.4 %)	18.40 ± 9.523 (3.3–59.4 %)	17.00 ± 6.388 (3–36 %)
**CD3^+^ CD8^+^** **T cell (%)**	40.92 ± 9.458 (14–69 %)	38.97 ± 11.88 (15–74 %)	39.93 ± 9.924 (14–71.09 %)
**CD3^+^ γδ T cell (%)**	5.380 ± 1.858 (1.84–10 %)	4.144 ± 4.412 (1–39 %)	5.183 ± 6.134 (0.62–49 %)
**SLEDAI**	3.767 ± 0.7508 (2–5)	5.072 ± 2.003 (2–14)	12.62 ± 4.849∗∗∗∗ (4-22)
**IgG (mg/dL)**	1928 ± 868.5 (1194–3887)	1419 ± 931.4 (397–4604)	1104 ± 847.8 (170.2–4540)
**anti-dsDNA (IU/mL)**	444.8 ± 313.2 (63.27–1161)	436.5 ± 319.9 (17.93–1400)	480.9 ± 365.4 (30.37–1589)
**C3 (mg/dL)**	72.85 ± 26.23 (26.4–120)	77.33 ± 24.48 (5.64–167.2)	70.42 ± 28.81∗∗ (5.55–176.0)
**C4 (mg/dL)**	12.53 ± 5.77 (2.170–23.70)	16.10 ± 10.61 (2.510–79.30)	13.61 ± 9.536∗∗ (1.680–63.40)
**WBC (K/μL)**	4.891 ± 1.570 (2.740–11.48)	6.401 ± 2.402 (0.450–13.49)	6.636 ± 3.619 (0.080–22.02)
**Hb (g/dL)**	11.58 ± 1.776 (5.600–14.60)	11.49 ± 1.770 (6.600–17.40)	10.67 ± 2.106∗∗∗∗ (5.200–17.90)
**Platelet (K/μL)**	273.2 ± 80.17 (71.00–475.0)	260.3 ± 109.6 (9.000–522.0)	268.8 ± 129.5 (10.00–699.0)
**ESR (mm/hr)**	21.95 ± 21.46 (2.000–89.00)	26.92 ± 23.16 (1.000–101.0)	30.98 ± 25.90 (1.000–140.0)
**CRP (mg/dL)**	0.2243 ± 0.5305 (0.02000–3.150)	0.4107 ± 1.018 (0.02000–9.180)	0.4172 ± 0.8755 (0.01000–6.020)
**serum Creatinine (mg/dL)**	0.4944 ± 0.1034 (0.3000–0.7000)	0.9509 ± 1.176 (0.01000–12.10)	1.127 ± 1.193∗∗ (0.3000–9.500)
**eGFR (mL/min/1.73 m**^**2**^**)**	209.5 ± 54.26 (106.7–347.0)	121.3 ± 60.37 (4.400–329.0)	120.6 ± 63.51 (6.700–271.4)

Active vs Inactive.

*: *P* < 0.05.

***: *P* < 0.001.

∗∗ : *P* < 0.01.

∗∗∗∗ : *P* < 0.0001.

### 2.2 Correlation between lymphocyte subsets and disease activity parameters

**Fig. 2:**
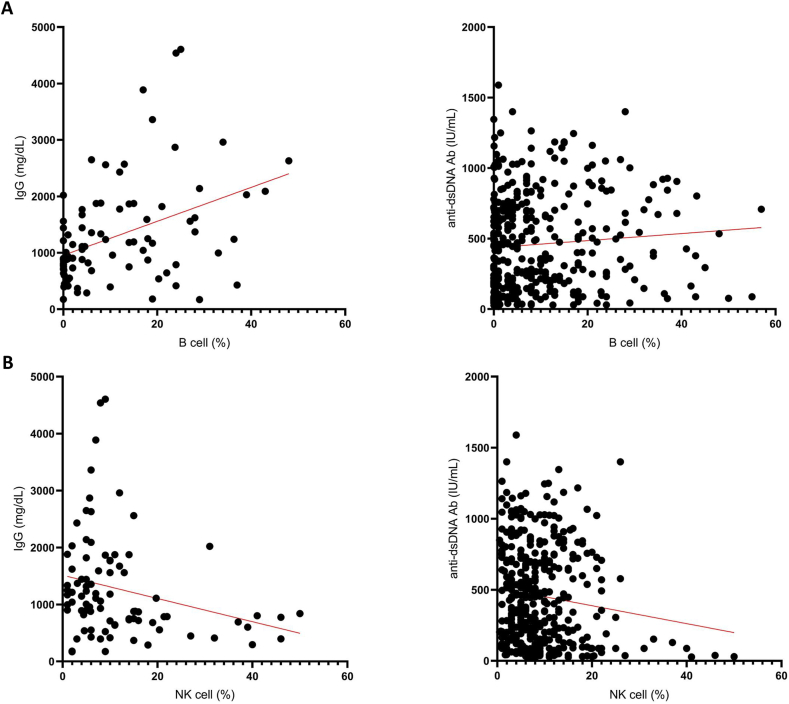

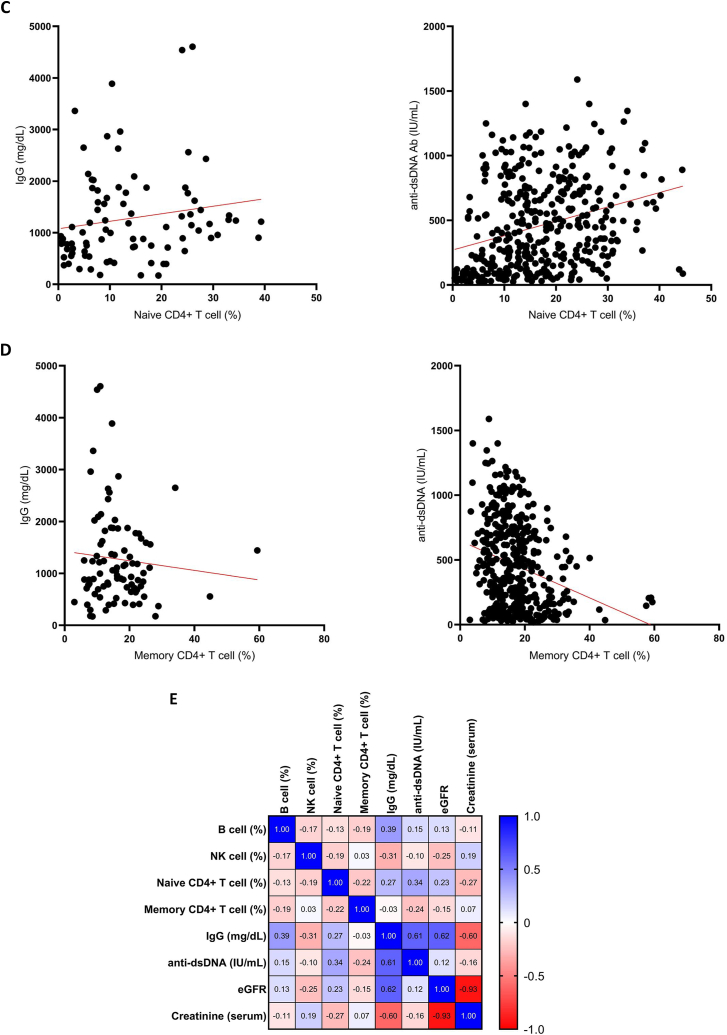
Relationship between the levels of IgG and anti-dsDNA antibodies in the serum and the percentages of B lymphocytes, natural killer (NK) cells, naive CD4^+^ T lymphocytes, and memory CD4^+^ T lymphocytes. The data in this figure was based on 455 clinical episodes where lupus nephritis (LN) was suspected. The relationship between the lymphocyte subset cells and IgG level (left) or anti-dsDNA Ab level (right)were correlated: (**A**) B lymphocytes, (**B**) natural killer (NK) cells, (**C**) naive CD4^+^ T lymphocytes, and (**D**) memory CD4^+^ T lymphocytes. Red solid lines represent correlation line. (**E**) Spearman's rank correlation matrix between lymphocyte subsets and selected parameters of LN. Correlations coefficient (*r*) were indicated in squares. The colors of the scale bar denote the nature of the correlation with +1 indicating perfect positive correlation (deep blue) and −1 indicating perfect negative correlation (deep red).

Next, we assessed the relationships between LN-associated parameters and lymphocyte subsets (Supplement Table 1). B cells were positively correlated with immunoglobulin G (IgG) and anti-dsDNA antibodies, whereas NK cells were negatively correlated with IgG levels. Memory CD4^+^ T cells were negatively correlated with anti-dsDNA levels, and naïve CD4^+^ T cells were positively correlated with both IgG and anti-dsDNA antibodies (Fig. 2A–E; Supplement Table 1).

### 2.3 Relationship between the mean percentages of lymphocyte subsets and longitudinal changes in eGFR

**Fig. 3:**
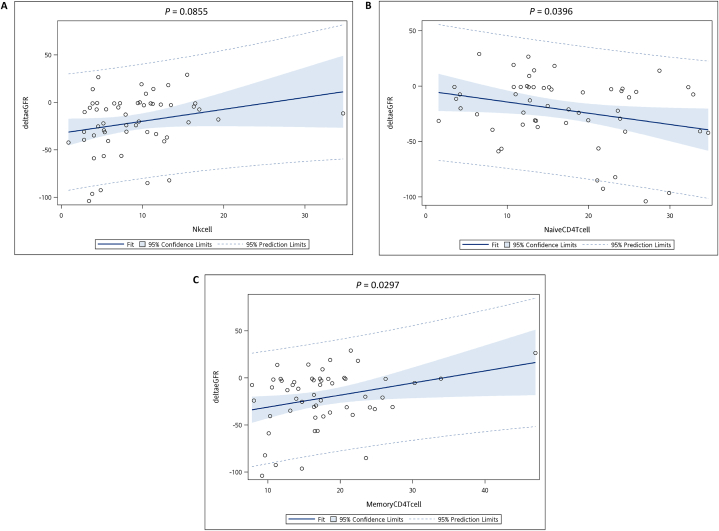
**Relationship between the change in eGFR (ΔeGFR) and the mean percentage of three types of lymphocyte subset cells (NK cells, naive CD4**^**+**^**T cells, and memory CD4**^**+**^**T cells) in 55 patients over a long period of time (more than 1 year).** The changes in kidney function was ΔeGFR as calculated by subtracting the starting eGFR value from the last eGFR value (delta eGFR, y-axis). The lymphocyte subset data from each patient were averaged as mean percentage (x-axis). The mean percentages of natural killer (NK) cells (**A**), naïve CD4^+^ T lymphocytes (**B**), and memory CD4^+^ T lymphocytes (**C**) were significantly correlated with ΔeGFR using the GLM procedures. These three subset cells were selected for further correlation analysis, Blue solid lines represent fitted line.

Evaluation of the relationship between the mean percentage of lymphocyte subsets and change in eGFR (ΔeGFR) over time revealed a positive correlation between NK and memory CD4^+^ T cells with ΔeGFR, whereas a negative correlation was observed between naïve CD4^+^ T cells and ΔeGFR (Fig. 3A–C). Furthermore, increased numbers of NK and memory CD4^+^ T cells showed a tendency for reduced eGFR deterioration, whereas that of naive CD4^+^ T cells showed an opposite trend (to deteriorate more).

We selected NK, naïve CD4^+^ T, and memory CD4^+^ T cells for further analysis based on their median–mean percentages, which correlated with ΔeGFR (*P* < 0.1; Supplement Table 2). A category score ranging from 0 to 3 for each patient was calculated using the following formula:

Score sum = NK cell category score (0 or 1) + naive CD4^+^ T cell category score (0 or 1) + memory CD4^+^ T cell category score (0 or 1)

**Fig. 4:**
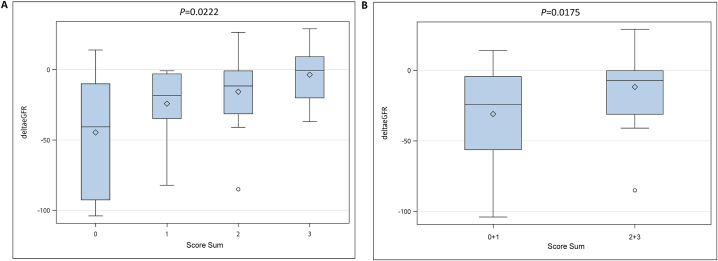
**Relationship between category of lymphocyte subset group and ΔeGFR.** We classified the 55 patients with long-term (>1 year) eGFR data into four groups based on their mean percentages of NK cells, naïve CD4^+^ T cells, and memory CD4^+^ T lymphocytes, respectively. The patients were assigned a score of 0 or 1 for each subset cell type, depending on whether their mean percentages of subset cell was above or below the median of subset cell. The category score for each patient was calculated by adding up the scores for the three subset cell types. The patients were then divided into four groups based on their category score: score sum = 0, 1, 2, or 3. (**A**) Box plot comparing changes in eGFR (ΔeGFR) across four different score sum groups (0, 1, 2, and 3). The x-axis represents the groups based on score sum, with each group representing a categorical variable with four values (0, 1, 2, 3). The y-axis represents the change in estimated glomerular filtration rate. This is a measure of kidney function, with negative values indicating a decline in eGFR. The mean ΔeGFR for the four patient groups was 44.65, 24.19, 15.69, and 3.656 mL/min/1.73 m2, respectively. (**B**) Box plot comparing changes in eGFR (ΔeGFR) across two combined score sum groups (0 + 1 and 2 + 3). The x-axis now shows only two categories, which represent the combined score sum groups: Group 1: combining score sum 0 and 1; Group 2: combining score sum 2 and 3. The central box represents the interquartile range (IQR), which contains the middle 50 % of the data for each group. The line within the box shows the median of the data. The diamonds represent the mean of the ΔeGFR for each group.

The patients with long-term (>1 year) eGFR data (n = 55) were divided into four groups based on the score sum: score sum = 0 (n = 9, ΔeGFR = −44.6500000 ± 43.3351907), score sum = 1 (n = 18, ΔeGFR = −24.1888889 ± 24.9386751), score sum = 2 (n = 19, ΔeGFR = −15.6947368 ± 25.4757940), and score sum = 3 (n = 9, ΔeGFR = −3.6555556 ± 22.1226756) (Supplement Table 3). The results showed significant differences among ΔeGFR across the four different score sum groups (0, 1, 2, and 3; Fig. 4A; ANOVA *P* = 0.0222, R^2^ = 0.170311).

We then combined the score sum = 0 and 1 groups into one group (Group 1) and the score sum = 2 and 3 groups into another (Group 2). These groups corresponded to the low NK-low memory CD4^+^ T-high naïve CD4^+^ T cell (Group 1) and the high NK-high memory CD4^+^ T-low naïve CD4^+^ T cell (Group 2), respectively (Supplement Table 3). The mean ΔeGFR of Group 1 (31.01 mL/min/1.73 m^2^) was significantly greater than that of Group 2(11.83 mL/min/1.73 m^2^; ANOVA *P* = 0.0175, R^2^ = 0.101890), suggesting a significantly reduced decline in eGFR in Group 2. This finding indicates that a specific composition of lymphocyte subsets can impair kidney function. Patients in Group 1 had a higher frequency of active LN episodes than those in Group 2 (Supplement Fig. 2). Overall, our results suggest that the specific composition of lymphocyte subsets plays a role in the progression of kidney diseases. The R^2^ value suggested that even though a significant relationship between the group scores and ΔeGFR was observed, some proportion of the variability in ΔeGFR remained unexplained by the score groups alone.

**Fig. 5:**
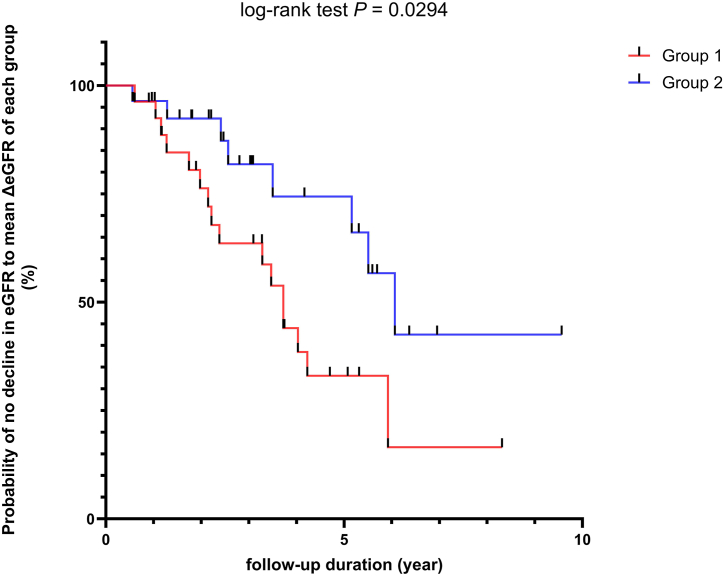
**Kaplan-Meier Analysis of eGFR Decline between Group 1 and Group 2 from 55 SLE patients.** There were two survival curves, one for each group. The red curve represents Group 1 (mean ΔeGFR 31.01 mL/min/1.73 m^2^), and the blue curve represents Group 2 (mean ΔeGFR 11.83 mL/min/1.73 m^2^). The x-axis represents the follow-up duration in years. The y-axis represents the probability of not experiencing the defined event (not having a decline in eGFR to the level of mean ΔeGFR for the group). Each step down in the curve represents the time point at which the event (decline in eGFR to the level of mean ΔeGFR) occurred for a patient. Vertical drops represent event occurrences. Tick marks (small vertical lines on the survival curves) indicate censored data, meaning that at those points, a patient was lost to follow-up or the study ended before the event occurred. The plot shows that Group 2 has a higher probability of not experiencing a decline in eGFR to their mean ΔeGFR level compared to Group 1. The difference in the curves indicates that Group 1 (low NK-low memory CD4^+^ T-high naïve CD4^+^ T group) had a faster decline in eGFR compared to Group 2 (high NK-high memory CD4^+^ T-low naïve CD4^+^ T group) (log-rank test *P* = 0.0294).

Next, we analyzed the probability of survival (not experiencing a decline in eGFR to their group mean ΔeGFR level) during follow-up periods, using the Kaplan–Meier method to investigate how the changes in eGFR over time differ between these two groups. Fig. 5 shows that Group 2 has a higher probability of survival than Group 1 (log-rank test *P* = 0.0294). Furthermore, 50 % of the patients in Groups 1 and 2 each exhibited ΔeGFR above the group mean ΔeGFR (11.83 and 31.01 mL/min/1.73 m^2^, respectively) at 5 and 10 years, respectively. Clinically, these findings suggest that patients in Group 2 had a slower progression to the mean decline in kidney function than those in Group 1, indicating potentially different risks between the two groups. Group 2 had a higher proportion of NK and memory CD4^+^ T cells, which protect against kidney function. In contrast, Group 1 had a higher proportion of naive CD4^+^ T cells, which are detrimental to kidney function. These findings were further supported by those of Kaplan–Meier analysis, which showed that the composition of lymphocyte subsets can affect changes in kidney function over time.

### 2.4 Differentially expressed genes (DEGs) involved in LN regulation and lymphocyte subset aberration

**Fig. 6:**
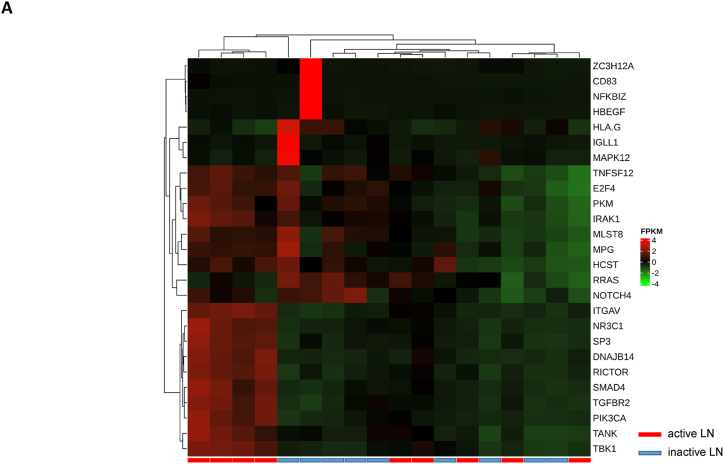

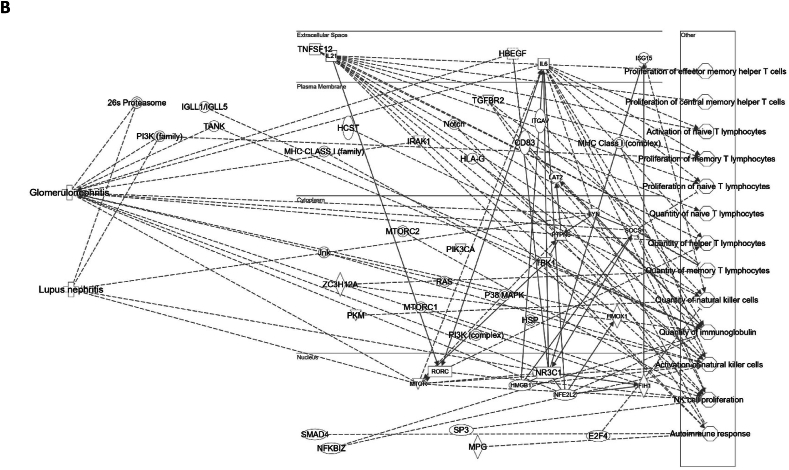

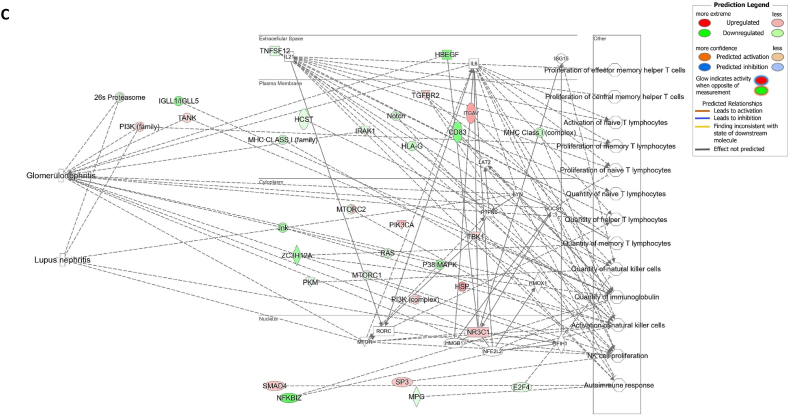

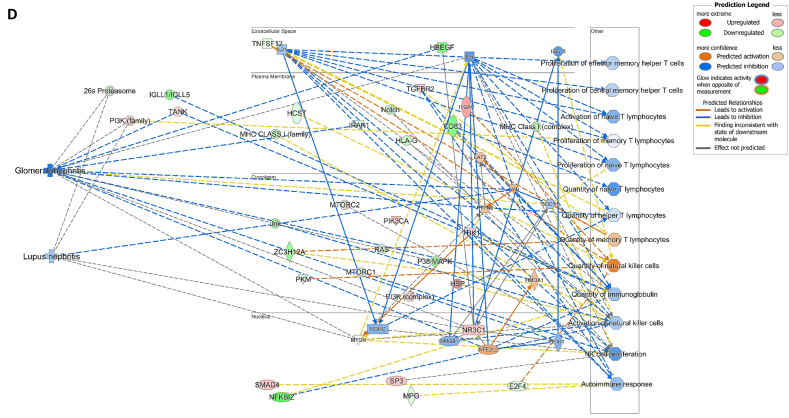

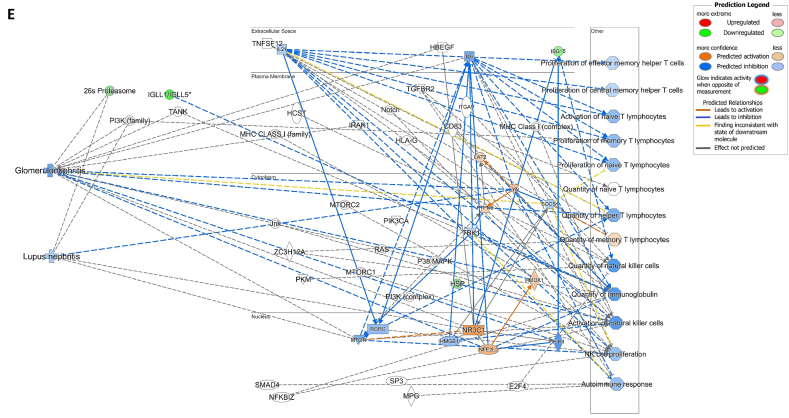

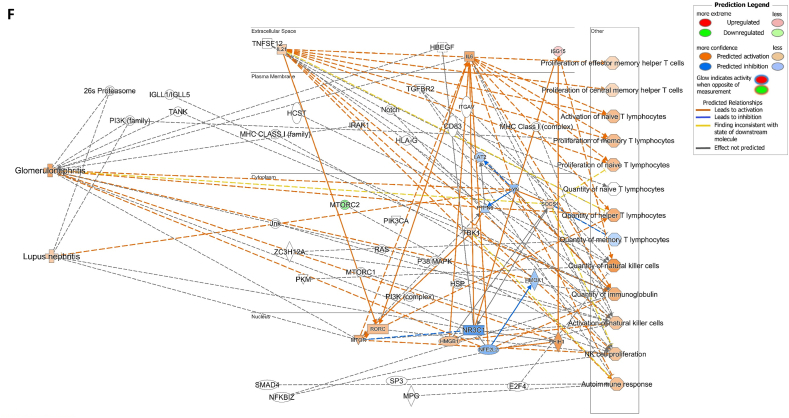

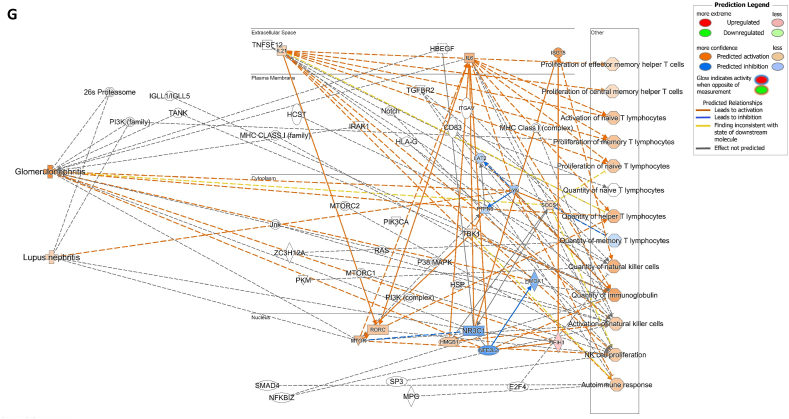
**Differentially expressed gene (DEG)-derived network regulating lymphocyte subset cell types, Glomerulonephritis (GN), and Lupus Nephritis (LN).** (**A**) A heatmap visualized the expression patterns of 28 DEGs related to GN and LN. Columns represent patients while rows depict mRNA genes. Upregulation was shown in red and downregulation in green. (**B**) The network visualized mRNA genes as nodes and their biological connections as edges. This network hadn't activated the MAP function, which predict downstream effects of upregulation or downregulation of molecules on disease/function. (**C**) DEGs relate to GN and LN were highlighted. The color of the mRNA gene node signified the level of upregulation (red) or downregulation (green). (**D**) DEG-derived network with the activated MAP function overlaid with mRNA data from 9 active LN vs 9 inactive LN patients. Predicted activation or increased expressions were in orange, while predicted inhibition or decreased expressions were in blue. Our DEG-derived network was anticipated to decrease the quantities of naive T lymphocytes and increase the quantities of both NK cells and memory T lymphocytes. Additionally, this network was projected to inhibit the status of both glomerulonephritis (GN) and lupus nephritis (LN). (**E**) DEG-derived network with the activated MAP function overlaid with mRNA data from GSE72747 test 2 dataset which compared 6 months after immunosuppressant treatment *vs* pre-treatment. The overall effect was increased memory T lymphocytes and suppressed GN and LN activity. (**F**) DEG-derived network with the activated MAP function overlaid with mRNA data from GSE 81622 test 1 dataset which compared LN patients *vs* normal control (NC). The overall effect was decreased memory T lymphocytes and activated GN and LN activity. (**G**) DEG-derived network with the activated MAP function overlaid with mRNA data from GSE 99967 test 2 dataset which compared SLE patients *vs* normal control (NC). The overall effect was decreased memory T lymphocytes and activated GN and LN activity.

We assessed gene expression in patients with active LN and compared it to that in patients with inactive LN. Using DESeq2, we identified 28 DEGs related to LN, glomerulonephritis (GN), naïve T lymphocytes, memory T lymphocytes, and NK cells (Table 3). The heat map of these 28 DEGs showed distinct gene expression patterns between patients with active and inactive LN (Fig. 6A). The DEG-derived network showed that these genes were interconnected in a complex manner (Fig. 6B–D). Ingenuity Pathway Analysis (IPA) predicted that the DEG networks promote the quantity of NK cells and memory T lymphocytes while inhibiting the number of naive T lymphocytes. Furthermore, the DEG network suppressed the activity of both GN and LN (Fig. 6D).

**Table 3:** Differentially expressed genes related to regulation of glomerulonephritis (GN) and lupus nephritis (LN).

Symbol	Entrez Gene Name	Expression (log2FoldChange)	Location	Family
*26s Proteasome complex*		−0.497	Cytoplasm	Complex
*CD83*	CD83 molecule	−3.14774	Plasma Membrane	transmembrane receptor
*E2F4*	E2F transcription factor 4	−0.49037	Nucleus	transcription regulator
*HBEGF*	heparin binding EGF like growth factor	−3.57431	Extracellular Space	growth factor
*HCST*	hematopoietic cell signal transducer	−0.67715	Plasma Membrane	transmembrane receptor
*HLA-G*	major histocompatibility complex, class I, G	−1.55851	Plasma Membrane	Other
*HSP*	DNAJB14	1.327	Cytoplasm	Group
*IGLL1/IGLL5*	immunoglobulin lambda like polypeptide 5	−3.06243	Plasma Membrane	Other
*IRAK1*	interleukin 1 receptor associated kinase 1	−0.44746	Plasma Membrane	Kinase
*ITGAV*	integrin subunit alpha V	1.105709	Plasma Membrane	transmembrane receptor
*Jnk*	MAPK8	−2.338	Cytoplasm	Group
*MPG*	N-methylpurine DNA glycosylase	−0.70755	Nucleus	Enzyme
*MTORC2*✽	RICTOR	1.134	Cytoplasm	Complex
*MTORC2*✽	MLST8	−0.630	Cytoplasm	Complex
*NFKBIZ*	NFKB inhibitor zeta	−4.29614	Nucleus	transcription regulator
*Notch*	NOTCH4	−1.12	Plasma Membrane	Group
*NR3C1*	nuclear receptor subfamily 3 group C member 1	0.698912	Nucleus	ligand-dependent nuclear receptor
*PI3K (complex)*		0.689333	Cytoplasm	Complex
*PIK3CA*	phosphatidylinositol-4,5-bisphosphate 3-kinase catalytic subunit alpha	0.865196	Cytoplasm	Kinase
*PKM*	pyruvate kinase M1/2	−0.60801	Cytoplasm	Kinase
*RAS*	RRAS	−0.941	Cytoplasm	Group
*SMAD4*	SMAD family member 4	0.722033	Nucleus	transcription regulator
*SP3*	Sp3 transcription factor	0.698179	Nucleus	transcription regulator
*TANK*	TRAF family member associated NFKB activator	0.491583	Cytoplasm	Other
*TBK1*	TANK binding kinase 1	0.558364	Cytoplasm	Kinase
*TGFBR2*	transforming growth factor beta receptor 2	0.599042	Plasma Membrane	Kinase
*TNFSF12*	TNF superfamily member 12	−0.44374	Extracellular Space	Cytokine
*ZC3H12A*	zinc finger CCCH-type containing 12A	−2.50818	Cytoplasm	Enzyme

∗ There were two protein gene associated with MTORC2: RICTOR and MLST8.

Next, we compared the DEGs identified in this study with those available in public datasets to validate our findings (Supplementary Table 4). Overlaying three public datasets (GSE72747, GSE81622, and GSE99967) onto our DEG-derived network revealed two types of network effects on lymphocyte subset composition and GN or LN status. As shown in Fig. 6D and **E**, most edges are blue, indicating inhibition relationships in the network from the present study (active vs. inactive LN) and GSE72747 (month six vs. pre-treatment). The overall effect was an increase in memory T lymphocytes and suppression of GN and LN activities. In contrast, Fig. 6F and **G** shows elevated numbers of orange edges, indicating more activation relationships in the networks from GSE81622 (LN vs. normal control) and GSE99967 (SLE vs. NC). The overall effect led to a decrease in memory T lymphocytes and an increase in GN and LN activities. Together, these data suggest that the DEG-derived network obtained in the present study is associated with the lymphocyte subset composition and GN or LN status.

**Fig. 7:**
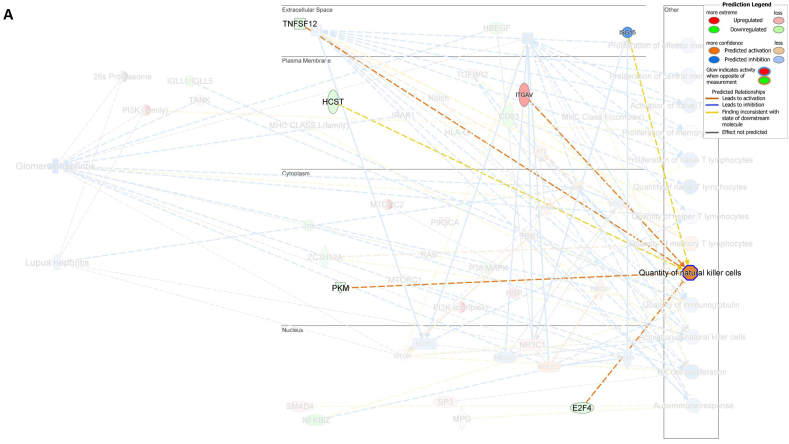

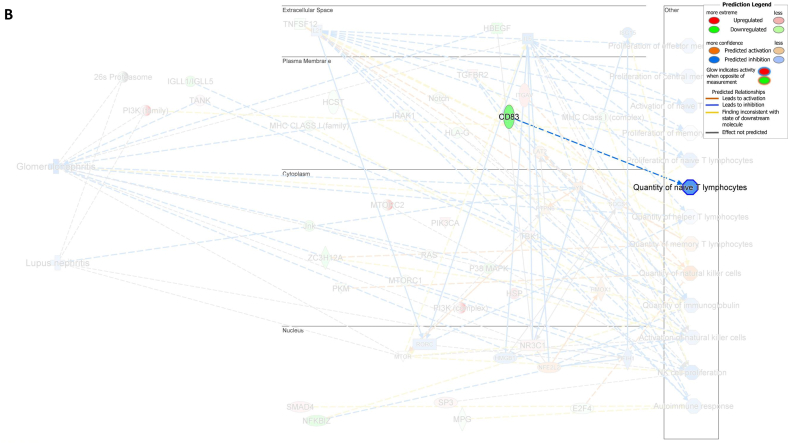

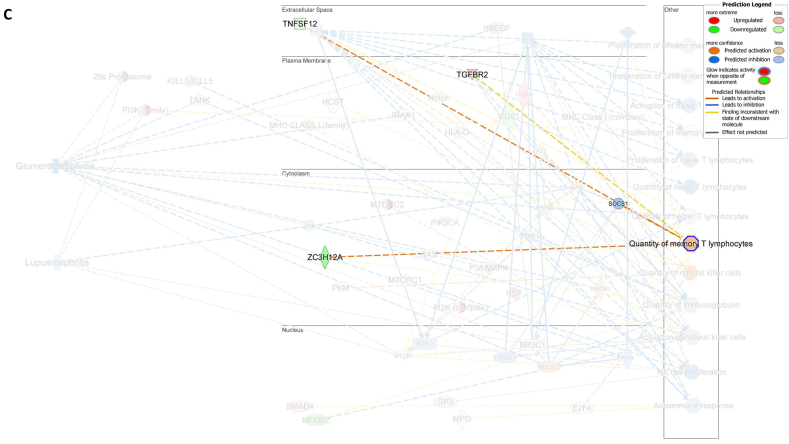

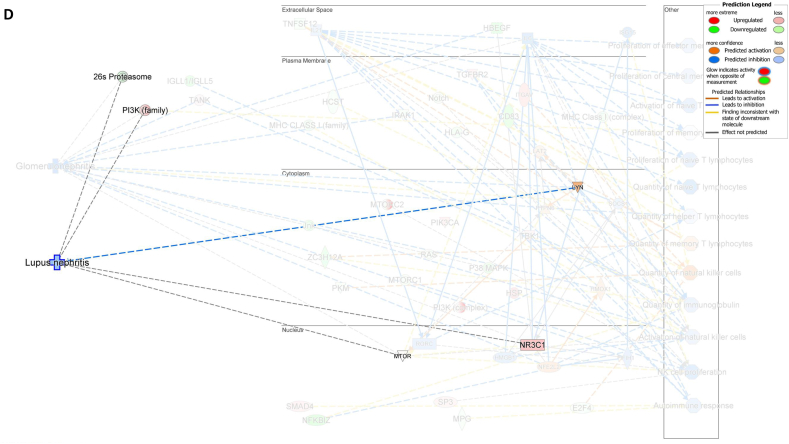

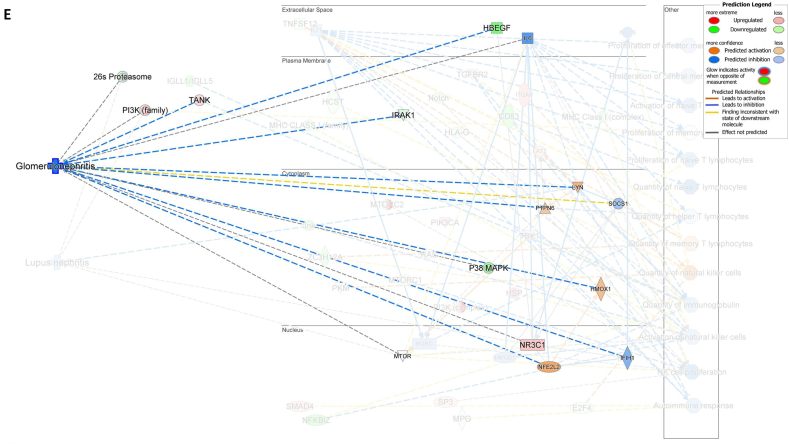
**Associations between differentially expressed genes (DEGs) in the molecular network with their predicted downstream impacts on lymphocyte subset cell types, Glomerulonephritis (GN), and Lupus Nephritis (LN).** (**A**) *E2F4* (downregulated), *ITGAV* (predicted activation), *TNFSF12* (downregulated), *PKM* (downregulated) led to increased quantities of NK cell. (**B**) Downregulated *CD83* led to decreased quantities of naive T lymphocyte. (**C**) *TNSSF12* (downregulated), *SOCS1* (predicted inhibition), and *ZC3H12A* (downregulated) led to increased quantities of memory T lymphocyte. (**D**) *LYN* (predicted activation) led to inhibition of LN. (**E**) *LYN* (predicted activation), *HMOX1* (predicted activation), *TANK* (upregulated), *HBEGF* (downregulated), *IRAK1* (downregulated), *PTPN6* (predicted activation), *IFIH1* (predicted inhibition), and *NFE2L2* (predicted activation) led to inhibition of GN. The color intensity of the mRNA gene node indicates the degree of either upregulation (red) or downregulation (green) of the respective mRNA. The color intensity of both the mRNA gene node and the disease/function node indicates the degree of predicted activity of either activated (orange) or inhibited (blue).

Among the genes that specifically regulate the NK cell quantity *E2F4*, *TNFSF12*, and *PKM* were downregulated while *ITGAV* was activated, leading to a decreased number of NK cells (Fig. 7A). Furthermore, the downregulation of *CD83* was found to be associated with a decrease in the number of naïve T lymphocytes (Fig. 7B). Additionally, the downregulation of *TNFSF12* and *ZC3H12A* and inhibition of *SOCS1* were found to be associated with an increased number of memory T lymphocytes (Fig. 7C). These findings demonstrated that the numbers of NK cells, memory T lymphocytes, and naïve T lymphocytes were regulated by the DEG-derived network.

The activation of *LYN*, *HMOX1*, *PTPN6*, and *NFE2L2* and the inhibition of *IFIH1* contributed to the inhibition of LN activity (Fig. 7D), whereas upregulation of *TANK* and downregulation of *HBEGF* and *IRAK1* led to inhibition of GN activity (Fig. 7E). These findings suggest that inhibition of DEG-derived networks plays a role in attenuating GN and LN, whereas its activation exacerbates GN and LN.

Together, our data showed that the DEG-derived network was associated with lymphocyte subset composition and the status of GN or LN. The findings also suggest that naïve CD4^+^ T lymphocytes play a detrimental role in LN and GN, whereas NK cells and memory CD4^+^ T lymphocytes play beneficial roles.

### 2.5 DEG-derived network and IFN signaling

**Fig. 8:**
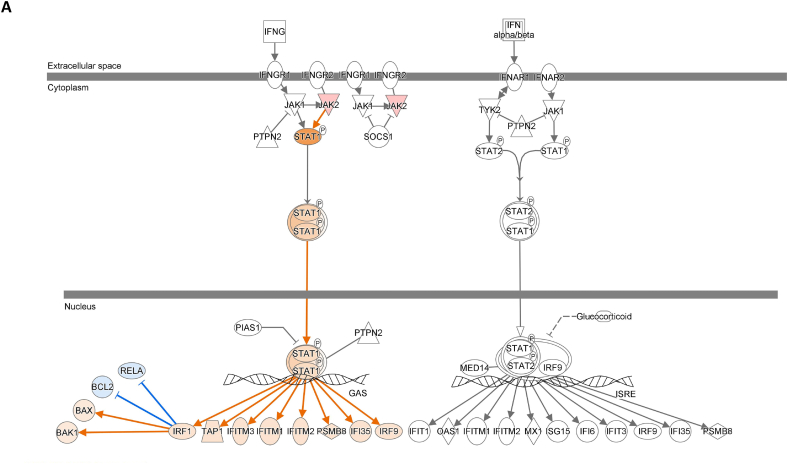

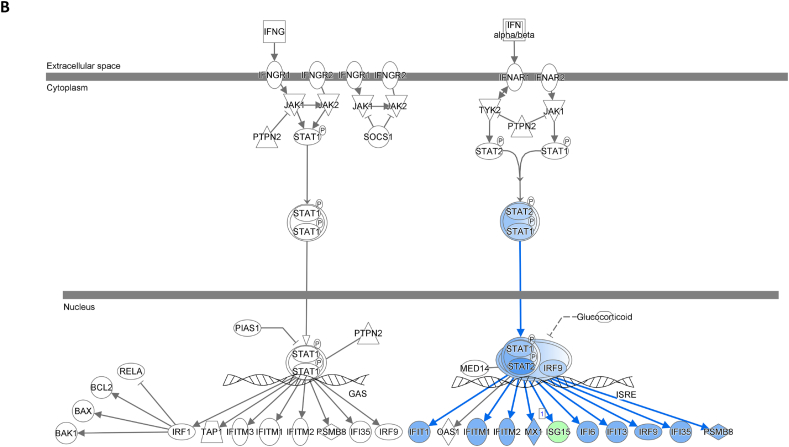

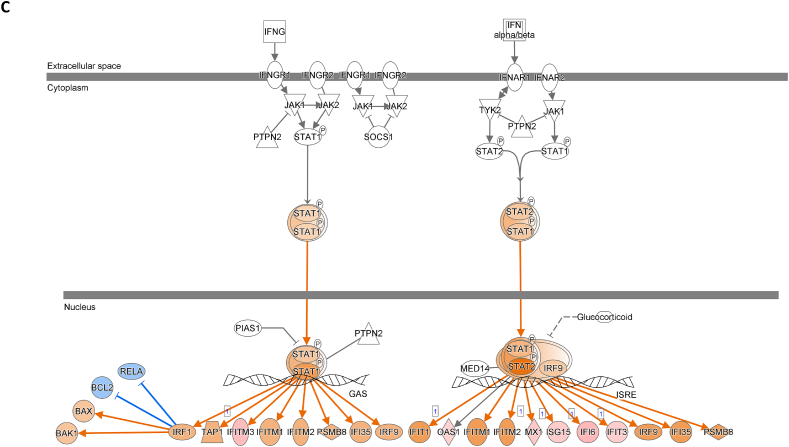

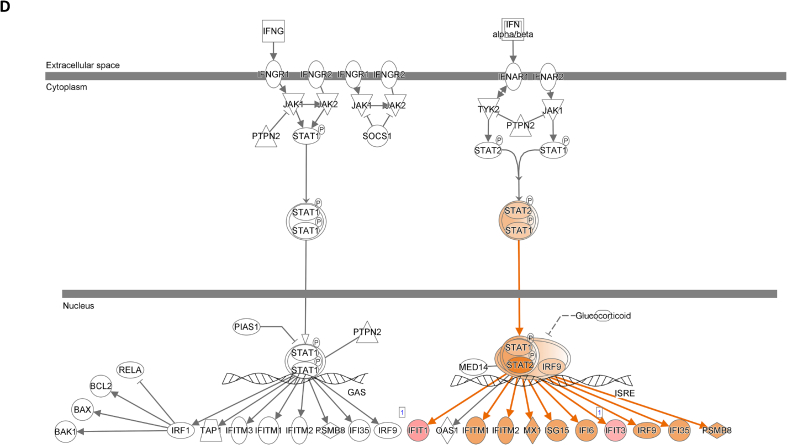
**Interferon signaling pathway involvement in SLE study.** DEG data of present study and from three GEO DEG data sets were applied onto the IFN signaling pathway from Ingenuity Pathway Analysis (IPA) database, respectively. The IFN-α/β pathway was shown in the right side while IFN-γ pathway was shown in the left side. (**A**) Application of DEG data of present study. (**B**) Application of DEG data of GSE72747 test 2. (C) Application of DEG data of GSE81622 test1. (D) Application of DEG data of GSE99967 test1. The color intensity of the mRNA gene node indicates the degree of predicted activity of either activated (orange) or inhibited (blue).

The interferon (IFN) signaling pathway plays a critical role in GN and LN. We used the "Pathways & Lists > Search" function in the Ingenuity Pathway Analysis (IPA) knowledge base to identify the IFN signaling pathway. We compared our DEG data with three publicly available DEG datasets (Fig. 8A–D; supplement Table 4) and overlaid all four datasets on the IPA IFN signaling pathway. The findings show that active LN increased IFN-γ activity compared to inactive LN (Fig. 8A). Concordantly, GSE81622 also showed increased IFN-γ activity in LN vs. normal control (Fig. 8C). Considering these results, we speculated that the increased IFN-γ activity (active vs. inactive LN) is associated with the increased number of NK cells predicted by our DEG-derived network.

## 3 Material and methods

### 3.1 Patients

The study population comprised 95 retrospectively enrolled patients with pediatric-onset SLE and renal involvement who presented with 450 clinical episodes (admission and outpatient clinics) and were suspected of having LN flares from December 1, 2011, to November 30, 2022. All patients were followed up every 2–3 months at the Department of Pediatrics, National Taiwan University Hospital (NTUH). Renal involvement and LN were defined according to the 1997 American College of Rheumatology (ACR) criteria for SLE and the International Society of Nephrology/Renal Pathology Society (ISN/RPS) 2003 classification to ensure consistency with widely recognized diagnostic standards [17]. Patients who did not meet the 1997 ACR criteria for SLE diagnosis and those with overlapping autoimmune diseases, other chronic inflammatory diseases, or malignancies were excluded. These exclusion criterion eliminate confounding factors that could have affected the study outcomes. Any episodes that fulfilled the definition of acute infections during follow-up were also excluded [18].

The following data were obtained during each follow-up or clinical episode: general laboratory tests for pediatric SLE, urinalysis, and PB lymphocyte subset quantification. Serum IgG levels were also measured to determine their correlation with disease activity [19]. SLEDAI-2K score, which is a modification of the original SLEDAI for proteinuria scoring, was used for clinical disease activity assessment in patients with SLE following a previous study [20]. Activity categories were defined based on the SLEDAI scores: no activity (SLEDAI = 0), mild activity (1–5), moderate activity (6–10), high activity (11–19), and very high activity (≥20) [21].

Suspected LN episode: The presence of LN was suspected in patients with SLE who developed active urinary sediment with persistent hematuria (≥5 RBCs, most of which were dysmorphic) or cellular casts, proteinuria, or an elevated serum level of creatinine (or a decrease in eGFR). Episodes that happened within three months after rituximab or steroid pulse therapy, with the potential of affecting the levels of both lymphocyte subsets and IgG, were excluded.

### 3.2 Outcomes definition

Following previous studies, the renal outcomes were defined in four categories [[22], [23], [24]]: **a**. *Improvement*: (i) 25 % increase of eGFR if the baseline eGFR is abnormal; (ii) at least 50 % reduction in the urinary protein-to-urinary creatinine ratio (UPCR) or at least 50 % reduction in urinary protein; (iii) a change from active urinary sediment (>5 RBCs/HPF and >5 WBCs/HPF or ≥1 cellular cast) to inactive urinary sediment (≤5 RBCs/HPF and ≤5 WBCs/HPF and no cellular casts). **b**. *No change*: Stable values for the eGFR or urinary protein. **c**. *Deterioration*: (i) 25 % decline in eGFR or end-stage kidney disease (ESKD); (ii) 100 % increase in UPCR; and (iii) presence of active urine in a subject whose urine was previously inactive. **d**. *Relapse*: (i) persistent urine protein >0.15 gm/day after complete remission or increase in proteinuria by > 1 gm/24 h, (ii) active urine sediment, or (iii) increase in serum creatinine by > 25 %.

Patients' episodes were classified into three groups: those not meeting the LN criteria according to ACR and ISN/RPS were categorized as non-LN episodes; those with outcomes **a** or **b** were deemed inactive LN episodes; and those with outcomes **c** or **d** were classified as active LN episodes.

Renal function was expressed as eGFR based on the modified Schwartz formula for pediatric and adolescent patients [25] and the Chronic Kidney Disease Epidemiology Collaboration (CKD-EPI) equation for adult patients [26]. The eGFR was calculated for each patient at each episode using a method validated for eGFR determination in patients with SLE [27]. The Kaplan–Meier method was used to estimate the patient and event-censored times of eGFR decline. The differences in the observed times for each subclass were assessed using the log-rank test.

### 3.3 Phenotyping of PB lymphocyte subsets in patients with/without LN using flow cytometry

A typical panel of markers, including CD3, CD4, CD8, CD19, and CD16^+^56, was used to identify the major lymphocyte subsets to assess immune dysregulation associated with SLE and LN. The freshly isolated and cultured PB lymphocytes were resuspended in PBS. To stain the surface antigens, cells were incubated with FITC-conjugated anti-CD3, APC-conjugated anti-CD4, PE-conjugated anti-CD8, APC-conjugated anti-CD19, or PE-conjugated anti-CD16^+^56 antibodies (all from BD Bioscience, USA). Mouse anti-human FITC-, PE-, and APC-conjugated IgG_1_ served as isotype controls. All cell samples were assayed using a FACSCalibur flow cytometer (BD Biosciences), and the acquired data were analyzed using the FCS Express V3 software (De Novo Software, Canada). The results were reported as percentages. The percentages and counts were determined for the following lymphocyte subsets: CD3^+^T, CD4^+^T, CD8^+^T, CD19^+^B, CD3^−^CD16^+^56NK, and CD3^+^CD16^+^56NKT cells. The CD4/CD8 ratio was then calculated. Human memory T cells were distinguished from naïve T cells based on their phenotypes (CD45RO^+^CD45RA^−^ and CD45RO^−^ CD45RA^+^, respectively; [28]).

Generalized estimating equation (GEE) analysis was performed using the GEE procedures in SAS software to compare lymphocyte subsets in different groups of patients with LN and analyze the correlation of those subsets with other parameters.

### 3.4 Classification of subset category responding to changes in the eGFR

Of the 95 patients, 55 with long-term (>1 year) eGFR data were selected to determine the impact of lymphocyte subset variation on renal function. Briefly, the mean percentages of lymphocyte subsets in each patient were calculated, and ΔeGFR was estimated as the difference between eGFR values of the first and last follow-up measurements. Subsequently, the correlation between the mean values of lymphocyte subsets and ΔeGFR was estimated in these 55 participants using the GLM method because of its ability to accommodate nonlinear relationships between lymphocyte subsets and renal function. The degrees of freedom, sum of squares, mean square, F-values, and *P*-values were calculated to determine the statistical significance of the correlations. The explanatory power of the model was assessed using R^2^ values (detailed reliability considerations are provided in the supplement information). The relationship between the mean values of lymphocyte subsets and ΔeGFR has been illustrated using fit plots, which include confidence and prediction limits to visualize the mean values of lymphocyte subset predictive capability and variability in ΔeGFR.

For further category analysis, NK, naïve CD4 T, and memory CD4 T with a *P* value of <0.1 were selected for further category analysis according to the significance between the mean values of lymphocyte subsets and ΔeGFR (Supplement Table 2). Categorical variables were then created by comparing the mean value of each participant's lymphocyte subset data with the median value of all participants' lymphocyte subset data to categorize them into high or low groups. For lymphocyte subsets that were positively correlated with ΔeGFR, a mean percentage of lymphocytes greater than the median was defined as a category with a score of 1, and a mean percentage of lymphocytes less than or equal to the median was defined as a category with a score of 0. Conversely, for lymphocyte subsets that were negatively correlated with ΔeGFR, a mean percentage of lymphocytes greater than the median was defined as a category with a score of 0, and a mean percentage of lymphocytes less than or equal to the median was defined as a category with a score of 1. Analysis of variance (ANOVA) was used to compare ΔeGFR in the different score sum groups.

The Kaplan–Meier analysis was used to estimate the survival function of two groups over follow-up time, with "survival" meaning the absence of a specific event.

### 3.5 Bulk RNA-seq to identify DEGs in active vs. inactive LN

Peripheral blood samples were obtained from randomly selected patients with active and inactive LN (n = 9 for each) adjusted for age and sex (supplement Table 5). Bulk RNA-seq of PB leukocytes was performed to investigate the transcriptional signatures associated with LN activity.

#### 3.5.1 Library preparation and sequencing

Purified RNA was used to prepare a sequencing library using the TruSeq Stranded mRNA Library Prep Kit (Illumina, San Diego, CA, USA) following the manufacturer's instructions. Briefly, mRNA was purified from total RNA (1 μg) on oligo(dT)-coupled magnetic beads and fragmented into small pieces under elevated temperature. First-strand cDNA was synthesized using a reverse transcriptase and random primers. After generating double-strand cDNA and adenylation of the 3′ ends of the DNA fragments, the adaptors were ligated, and then the samples were purified using the AMPure XP system (Beckman Coulter, Beverly, USA). Library quality was assessed using an Agilent Bioanalyzer 2100 system and a real-time PCR system. Qualified libraries were sequenced on an Illumina NovaSeq 6000 platform, with 150 bp paired-end reads generated by Genomics, BioSci & Tech Co. (New Taipei City, Taiwan).

#### 3.5.2 DEG analysis

Low-quality bases and adaptor sequences were removed from the raw data using FASTP (version 0.20.0). Filtered reads were aligned to the reference genome using HISAT2 (version 2.1.0). The software FeatureCounts (v2.0.1) in the Subread package was used to quantify gene abundance. DEGs were identified using DESeq2 (version 1.28.0) or EdgeR (version 3.36.0) [29,30], depending on the presence or absence of biological replicates.

### 3.6 IPA

Ingenuity Pathway Analysis was employed to integrate DEG data with existing biomedical knowledge databases to investigate the key pathways and gene networks involved in LN pathogenesis. IPA helps mitigate the challenges of studies with small sample sizes by leveraging its vast database to draw meaningful biological interpretations and predictions from DEGs.

#### 3.6.1 Network construction using relevant DEGs

We used the "Disease & Function Search" function of IPA to find molecules related to GN, LN, and quantities of naive T lymphocytes, memory T lymphocytes, and NK cells in the IPA Ingenuity knowledge base.

#### 3.6.2 Core analysis

A cutoff of log_2_ fold change (log_2_FC) was applied before the core analysis to focus the analysis on the top DEGs, which ensured that the magnitude of the change in the expression of the genes of interest was sufficiently strong. Filters were applied during the core analysis to limit the number of molecules in the dataset.

IPA supplies >700 predefined “canonical pathways,” detects pathway overlaps in significant dataset molecules, and predicts whether these pathways are activated or inhibited. IPA was performed using an algorithm that compared the upregulated and downregulated DEGs in the data with the pattern expected for the pathway if it were activated.

#### 3.6.3 Molecule activity predictor (MAP)

The MAP tool enables the prediction of the upstream or downstream effects of the activation or inhibition of molecules in a network or pathway, given one or more neighboring molecules with known activity. Thus, it allows the visualization of the overall effect of a pathway or network and, therefore, the formulation of a more accurate hypothesis. Based on the algorithm underlying the MAP tool (https://qiagen.my.salesforce-sites.com/KnowledgeBase/articles/Knowledge/MAP-Molecule-Activity-Predictor), molecules or functions predicted to be activated or inhibited are depicted in different colors according to their interactions with molecules known to be activated or inhibited. The principle of the algorithm is that predictions made for molecules adjacent to expressed molecules have a relatively high confidence value depending on the consistency of the findings supporting their activity direction. Conversely, molecules away from the expressed molecules show lower confidence values. Activity is assumed to be at the protein level for the nodes in a network or pathway. In this study, increased or decreased expression referred to increased or decreased protein activity, respectively.

## 4 Discussion

The study investigated lymphocyte subsets and gene expression profiles in patients with LN, revealing distinct patterns associated with disease activity. Patients with active LN showed reduced levels of NK and memory CD4^+^ T cells, while levels of naive CD4^+^ T cells were increased. These lymphocyte subset changes were correlated with IgG levels and renal function decline. Additionally, a predictive model based on DEGs suggested a regulatory effect on GN and LN activities. The study highlights the potential of these findings for developing biomarkers and therapeutic targets in LN.

T-cell abnormalities in LN have been extensively studied, showing a reduction in total CD4^+^ T cell frequencies [31]. A previous study has reported increases in memory CD4^+^ T cells and decreases in naïve CD4^+^ T cells in SLE [14]. However, pediatric LN patients exhibit increased frequency of effector memory CD4^+^CD45RO^+^CCR7^−^ T cells and reduced naïve CD4^+^CD45RA^+^CCR7^+^ T cells [32]. Consistent with previous studies, the present study demonstrated that the overall effect of DEG networks was involved in the promotion of the quantities of memory T lymphocytes and inhibition of those of naïve T lymphocytes.

As SLE activity increases, the serum levels of anti-dsDNA antibody levels typically increase, whereas those of complement C3 and C4 decrease, often leading to clinical deterioration of kidney function. The binding of dsDNA to the glomerular basement membrane (GBM) results in the formation of anti-dsDNA–dsDNA immune complexes, and their deposition along the GBM and elevated anti-dsDNA antibody levels are indicative of active LN [4]. Furthermore, patients with autoimmune diseases, such as SLE, exhibit hypergammaglobulinemia, which may correlate with disease activity [19]. In our study, IgG/anti-dsDNA Ab levels were positively correlated with the number of naïve CD4^+^ T lymphocytes and negatively correlated with those of NK cells and memory CD4^+^ T lymphocytes. This could explain the observed decreasing trend in ΔeGFR in parallel with the changes in the mean percentage of naïve CD4^+^ T cells, contrasting with the stable or increasing trend in ΔeGFR with changes in the mean percentages of NK and memory CD4 + T cells. However, the contribution of innate immune cells, particularly NK cells, remains unclear. Studies have shown that low NK cell counts are associated with LN [33,34]. In this study, we showed increased IFN-γ production on PB NK cells in patients with active SLE and LN. The reduced number of circulating NK cells can be attributed to the migration of highly cytotoxic NK cells from the PB to the kidneys in patients with LN and the consequent damage to local tissue [35]. Furthermore, the kidney immune cell profile of patients with SLE has recently been established using single-cell RNA-seq, which showed that PB NK cells acquired anti-inflammatory activities [13]. While NK cells could contribute to renal inflammation and damage in LN through their IFN-γ production, they could also be part of a compensatory immune response aiming to regulate or contain the inflammation [35]. Coit et al. demonstrated that naive CD4^+^ T cells in lupus undergo an epigenetic proinflammatory shift, implicating effector T cell responses in lupus flare [36] supporting the harmful role of naive CD4^+^ T lymphocytes.

During the development and progression of SLE, CD3^+^ γδ T cells play an important role due to their ability for antigen presentation, proinflammatory cytokines secretion, and promotion of antibody production by supporting B cells [37]. However, the percentage of CD3^+^ γδ T cells was remarkably decreased in patients with active SLE compared to that in those with inactive SLE, and the count of these cells showed an inverse correlation with disease activity [38]. Consistent with these findings, the present study also revealed a decrease in the number of CD3^+^ *γδ* T cells in patients with active LN and showed that CD3^+^ *γδ* T cell numbers were inversely proportional to the anti-dsDNA level (Supplement Table 1).

In a study by Żabińska et al., the percentage of CD3^+^CD8^+^CD28 cells was significantly higher in patients with SLE than that in healthy controls. Moreover, the study also showed that the number of these cells was positively correlated with disease activity, suggesting that CD3^+^CD8^+^CD28 cells play a pathogenic role in SLE, possibly by supporting autoantibody production [39]. Our study demonstrated similar findings, with an increased number of CD3^+^CD8^+^ T cells in patients with active LN, further supporting the role of CD3^+^CD8^+^ T cells in SLE pathogenesis.

The overexpression of type I IFN-related genes in PB immune cells is a key characteristic of SLE. Several studies have shown that IFN-α is dominant in SLE, however, some studies have suggested that the type II IFN-γ gene signature may occur early in SLE and play an important role in LN [40]. Serum levels of IFN-γ in patients with SLE were higher than those in patients without LN and healthy controls [41]. Fava et al. demonstrated that patients with LN could be stratified based on their IFN-γ-inducible chemokine levels, with higher levels associated with proliferative LN [42]. IFN-γ is a pleiotropic type II IFN that is mainly produced by effector Th1 CD4^+^ T, cytotoxic CD8^+^ T, and NK cells. Activated NK cells could be among the main IFN-γ producers responsible for the inflammation in LN [43]. IFN-γ can promote the differentiation of naive CD4^+^ T cells into inflammatory Th1 and Th17 cells. In this study, we showed that the overall effect of the DEG-derived network was to increase the number of NK cell types (Fig. 6). Type I IFN (IFN-α/β) has been shown to increase the count of CD56^+^ NK cells [44]. Our findings revealed that active LN was associated with increased IFN-γ activity. Based on these findings, we speculated that the increased number of NK cells predicted by our DEG-derived network may have led to increased IFN-γ activity.

*Lyn* deficiency exacerbates nephritis and arthritis in mice [45], while *HMOX1* upregulates the expression of glomerular decay-accelerating factors and minimizes complement deposition and injury [46]. *TANK* is a negative regulator of Toll-like receptor signaling and is critical for the prevention of autoimmune nephritis [47]. *HBEGF* promotes glomerular injury and renal failure in rapidly progressive crescentic glomerulonephritis [48]. Suppression of *IRAK1* catalytic activity has been shown to prevent LN in mice [49], whereas *PTPN6* ablation promotes Th1 cell differentiation and induces autoimmunity [50]. *IFIH1* is reported to be associated with *MDA5*-mediated chronic type I IFN gene signature and accelerated autoimmunity [51]. *NFE2L2* suppresses LN through inhibition of oxidative injury and the *NF-κB*-mediated inflammatory response [52]. In this study, we performed IPA based on an ingenuity knowledge base (expert-curated information from the literature) and integrated public databases (Entrez Gene, OMIM, GO, KEGG, Clinical trials, etc.) for network construction and functional analysis. The DEG-derived network revealed the predicted activation of *LYN*, *HMOX1*, *PTPN6*, and *NFE2L2*. It also predicted the inhibition of *IFIH1*, upregulation of *TANK,* and downregulation of *HBEGF* and *IRAK1*. All these factors inhibit GN. Together, these findings suggest the potency of these genes as biomarkers or therapeutic targets against LN; however, further experiments are required to validate our speculations.

We acknowledge the intricate nature of LN pathogenesis and its diverse impacts on disease progression [4,53]. While our findings are preliminary, we aim to stimulate further research in this area. Our study primarily focused on the role of lymphocyte subsets in LN progression; however, future research should include a comprehensive analysis of additional contributing factors. This could involve conducting multivariate analyses to distinguish the effect of lymphocyte subsets from other influential factors, such as variations in immunosuppressive therapy, infection history, genetic background, environmental factors, sample size and diversity, as well as longitudinal dynamics. Our study had some limitations. It involved 95 participants, which, while adequate for initial observations, would require a larger cohort for generalization of the findings. Due to its cross-sectional nature, this study cannot conclusively establish causality between lymphocyte subset composition and the progression of LN. Long-term eGFR data were available for only 55 patients. A more consistent longitudinal approach would enhance the strength of our findings. We validated our DEGs using publicly available datasets, and independent replication in different cohorts further confirmed the robustness of the predictive capacity of the DEG-derived network. Bioinformatics analyses, primarily speculative, were intended to guide future studies and inform further functional experiments for validation. Our study was primarily observational. Functional studies are required to elucidate the mechanistic roles of identified lymphocyte subsets and DEGs in LN pathology.

In conclusion, we established a significant link between specific lymphocyte subsets, DEG networks, and LN pathogenesis. Patients with active LN episodes exhibited a distinct lymphocyte profile characterized by reduced NK and memory CD4^+^ T cells compared with those with inactive LN. Moreover, NK and memory CD4^+^ T cell counts demonstrated a negative correlation with IgG levels, whereas naïve CD4^+^ T cells exhibited a positive correlation with IgG and anti-dsDNA levels. Lymphocyte subsets were correlated with ΔeGFR, suggesting that NK and memory CD4^+^ T cells might play a protective role, whereas naive CD4^+^ T cells might have a detrimental impact on renal function progression. Our scoring model enabled us to predict the decline in kidney function over time, with significant implications for clinical prognosis. Our DEG-derived network, validated using external (GEO) datasets, was predictive of the lymphocyte subset composition and had a regulatory effect on GN and LN activities. Our study highlighted the enhanced IFN-γ activity in active LN, potentially offering a molecular target for therapeutic interventions.

## Funding

The authors declare that they have no funding

## Data availability

The datasets generated and/or analyzed during the current study are available in Gene Expression Omnibus (GEO) series accession number GSE232381.

## Ethics approval and consent to participate

This study was approved by the Institutional Review Board and Research Ethics Committee of the National Taiwan University Hospital (Ethical Number: 202106098RINC) and was conducted in compliance with the protocol for good clinical practices and the principles of the Declaration of Helsinki. Informed consent was obtained from all participants and/or their legal guardians.

## CRediT authorship contribution statement

**Yi-Chen Chen:** Writing – original draft, Validation, Software, Methodology. **Hsin-Hui Yu:** Validation, Resources, Data curation. **Ya-Chiao Hu:** Validation, Resources, Data curation. **Yao-Hsu Yang:** Validation, Resources, Data curation. **Yu-Tsan Lin:** Validation, Resources, Data curation. **Li-Chieh Wang:** Validation, Resources, Data curation. **Bor-Luen Chiang:** Validation, Supervision, Resources, Data curation. **Jyh-Hong Lee:** Writing – review & editing, Writing – original draft, Visualization, Validation, Supervision, Methodology, Investigation, Formal analysis, Conceptualization.

## Declaration of competing interest

The authors declare that they have no known competing financial interests or personal relationships that could have appeared to influence the work reported in this paper.
