# Supplementary material for: Peripheral blood cells RNA-seq identifies differentially expressed gene network linked to lymphocyte subsets alterations and active lupus nephritis associated with declines in renal function
Source: Heliyon. 2024 Jun 2;10(11):e32303. doi: 10.1016/j.heliyon.2024.e32303 (PMC11190669; doi:10.1016/j.heliyon.2024.e32303)
Supplement: Multimedia component 1 [file mmc1.docx]

**Peripheral Blood Cells RNA-seq Identifies Differentially Expressed Gene Network Linked to Lymphocyte Subsets Alterations and Active Lupus Nephritis associated with Declines in Renal Function**

Yi-Chen Chen, Hsin-Hui Yu, Ya-Chiao Hu, Yao-Hsu Yang, Yu-Tsan Lin, Li-Chieh Wang, Bor-Luen Chiang, Jyh-Hong Lee^*^

**Statistical Reliability Considerations of GLM analysis for ΔeGFR versus four different scores sum group (0, 1, 2, and 3)**

A. Reliability Considerations of **Figure 4A**

**Statistical Significance of ANOVA Results:** The analysis identified significant differences in ΔeGFR across the scores, as evidenced by the ANOVA results with an F-value of 3.49 and a *P* value of 0.0222. This suggested that the treatment or condition associated with these scores affects kidney function. This support the reliability of the groupings based on scores.

**Parameter Estimates:** Estimates for the intercept and each score level (0, 1, 2) were provided along with their standard errors and 95% confidence intervals. The intercept and score group had specific estimates, indicating the expected change in ΔeGFR for each score compared to the reference (Score 3).

| Parameter | Estimate | Std Error | 95% CI | |
| --- | --- | --- | --- | --- |
| Intercept | −3.65555556 | 9.47350847 | −22.67441740 | 15.36330629 |
| Score 0 | −40.99444444 | 13.39756416 | −67.89117681 | 14.09771208 |
| Score 1 | −20.53333333 | 11.60263091 | −43.82658684 | 2.75992017 |
| Score 2 | −12.03918129 | 11.50040309 | −35.12720389 | 11.04884132 |
| Score 3 | 0.00000000 | . | . |  |

**Model Fit and Effect Size:** R-squared Value = 0.170311. This value indicated that approximately 17.03% of the variance in ΔeGFR is explained by the model, specifically the differences in scores (0, 1, 2, and 3). While significant, an R-squared value of around 17% suggested that some proportion of the variability in ΔeGFR remained unexplained by the score groups alone.

B. Reliability Considerations of **Figure 4B**

**Statistical Significance of ANOVA Results:** The model's F-value was 6.01 with a *P* value of 0.0175, indicating a statistically significant difference between the two groups in their effect on ΔeGFR. This suggested that the grouping by Score was meaningful in explaining changes in kidney function.

**Parameter Estimates:** The parameter estimates indicated how changes in Score grouping relate to changes in ΔeGFR. The significant coefficient for Score group 0+1 (-19.18425926 with a *P* value of 0.0175) suggested that belonging to this group was associated with a notable decrease in ΔeGFR compared to Score group 2+3.

**Model Fit and Effect Size:** The R-squared value 0.101890 indicated that approximately 10.19% of the variability in ΔeGFR was explained by the model. This R-squared value suggested that while there was some relationship between the scores and ΔeGFR, some portion of the variance in ΔeGFR was left unexplained by the model.

**Supplement Figure 1.** Representative diaphragm of flow cytometry analysis between active LN and inactive LN.


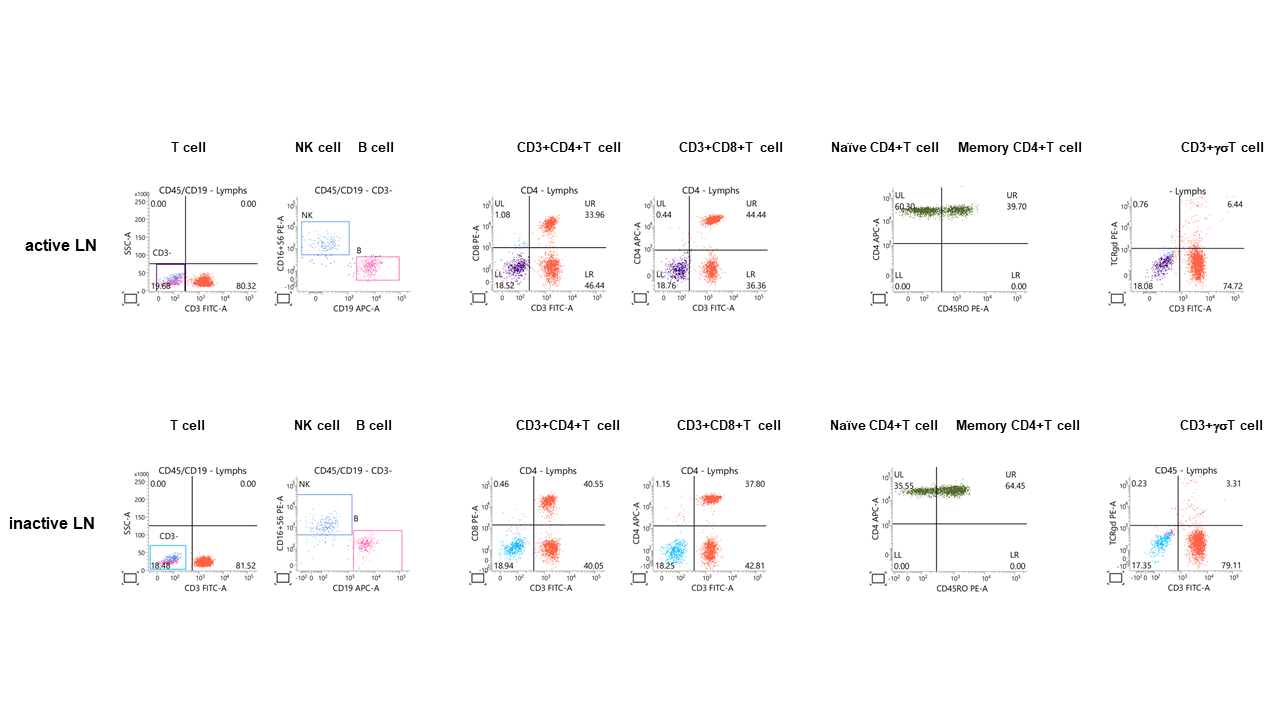


**Supplement Figure 2.** Comparison of episodes of non-LN, inactive LN, and active LN between score sum Group 1 and Group 2. Episodes of active LN was more frequent in patients within score sum Group 1 than in those within Group 2. Episodes of inactive LN was more frequent in patients within score sum Group 2 than in those within Group 1.


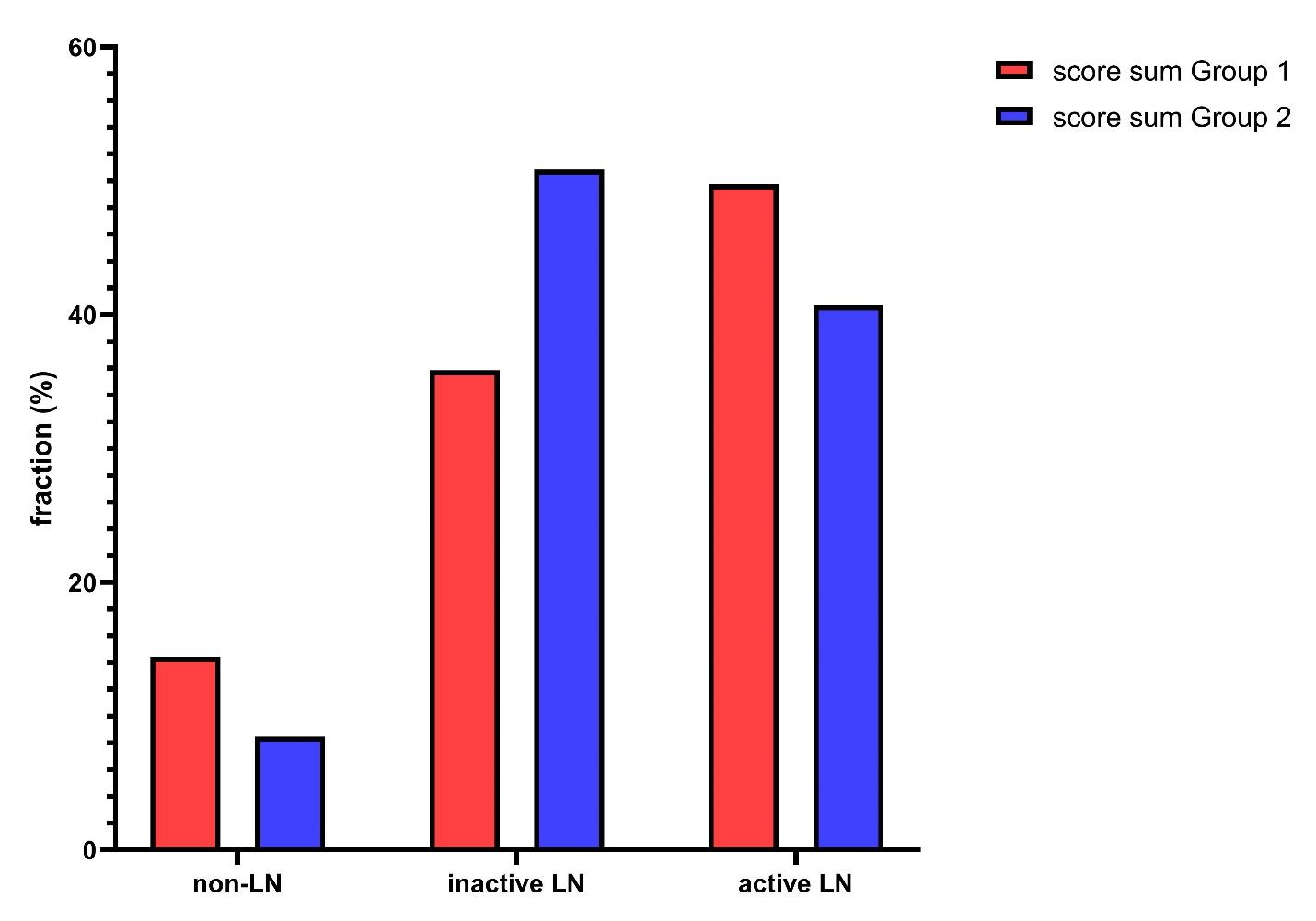


**Supplement Table 1.** Correlation matrix showing Spearman’s rank correlation coefficient *r* between lymphocyte subset and disease activity related parameters.

|  | SLEDAI | IgG | anti-dsDNA | C3 | C4 | ESR | eGFR | creatinine (serum) |
| --- | --- | --- | --- | --- | --- | --- | --- | --- |
| T cell (%) | -0.04253 | -0.01746 | -0.0427 | 0.1304  ** | 0.1008  * | -0.08873 | 0.06611 | -0.07042 |
| B cell (%) | -0.01081 | **0.3911**  *** | 0.1489  ** | -0.1586  *** | **-0.2607**  **** | 0.08203 | 0.134  ** | -0.1093  * |
| NK cell (%) | -0.00632 | **-0.3091**  ** | -0.09834  * | 0.0827 | 0.1707  *** | 0.03392 | **-0.2532**  **** | 0.1897  **** |
| Total  CD4+ T cell (%) | -0.07079 | 0.1527 | 0.1070  * | -0.006 | -0.01334 | 0.1452  ** | 0.1269  ** | **-0.2227**  **** |
| Naive  CD4+ T cell (%) | 0.002633 | **0.2696**  * | **0.3434**  **** | -0.05474 | -0.05524 | 0.1358  ** | **0.2327**  **** | **-0.2736**  **** |
| Memory  CD4+ T cell (%) | -0.07789 | -0.03096 | **-0.2435**  **** | 0.09888  * | 0.07336 | 0.0545 | -0.1455  ** | 0.06803 |
| CD3+CD8+ T cell (%) | 0.0506 | -0.1918 | -0.02597 | 0.1018  * | 0.1077  * | -0.09219 | -0.06282 | 0.0958  * |
| CD3+ γσ T cell (%) | -0.05697 | 0.08293 | -0.1142  * | 0.1567  *** | 0.07236 | **-0.2678**  **** | 0.181  *** | -0.08223 |

*: *P*<0.05; **: *P*<0.01; ***: *P*<0.001; ****: *P*<0.0001.

For absolute values of *r*, 0-0.19 is regarded as very weak, 0.2-0.39 as weak, 0.40-0.59 as moderate, 0.6-0.79 as strong, and 0.8-1 as very strong correlation.

**Supplement Table 2.** Statistical *P* values value for correlation between the mean values of lymphocyte subset and ΔeGFR

| subset | T cell (%) | B cell (%) | NK cell (%) | Total CD4 T cell (%) | Naive CD4 T cell (%) | Memory CD4  T cell (%) | CD3+ CD8+  T cell (%) | CD3+ gamma/delta T cell (%) |
| --- | --- | --- | --- | --- | --- | --- | --- | --- |
| P value | 0.6745 | 0.4079 | 0.0855 | 0.8155 | 0.0396 | 0.0297 | 0.9020 | 0.3322 |

**Supplement Table 3.** Composition of score sum added by score value of NK cells, naïve CD4+ T cells and memory CD4+ T cells.

|  | NK  category | Naïve CD4 category | Memory CD4 category |
| --- | --- | --- | --- |
| Score sum = 0 group (n=9, ΔGFR = -44.6500000 ± 43.3351907) | | | |
|  | 0 | 0 | 0 |
| Score sum =1 group (n= 18, ΔGFR =-24.1888889 ± 24.9386751) | | | |
|  | 1 | 0 | 0 |
|  | 0 | 1 | 0 |
|  | 0 | 0 | 1 |
| Score sum = 2 group (n=19, ΔGFR = -15.6947368 ± 25.4757940) | | | |
|  | 1 | 1 | 0 |
|  | 1 | 0 | 1 |
|  | 0 | 1 | 1 |
| Score sum = 3 group (n=9, ΔGFR = -3.6555556 ± 22.1226756) | | | |
|  | 1 | 1 | 1 |

For NK cell and memory CD4+ T cell, “0” represent mean percentage < median=; “1” represent mean percentage > median.

For naïve CD4+ T cell, “0” represent mean percentage > median=; “1” represent mean percentage < median.

|  | NK  category | Naïve CD4  category | Memory CD4  category |
| --- | --- | --- | --- |
| Score sum = 0 group | | | |
|  | low | high | low |
| Score sum = 1 group | | | |
|  | high | high | low |
|  | low | low | low |
|  | low | high | high |
| Score sum = 2 group | | | |
|  | high | low | low |
|  | high | high | high |
|  | low | low | high |
| Score sum = 3 group | | | |
|  | high | low | high |

“low” represent mean percentage < median; “high” represent mean percentage > median.

**Supplement Table 4.** Comparing our DEG data with GEO DEG data sets by IFN pathway analysis.

|  | NA# | Source | Comparison | IFN-γ pathway | IFN-α/β pathway |
| --- | --- | --- | --- | --- | --- |
| Current network |  | PB | A vs I | activated | Uninfluenced |
| GSE72747.gpl570.test2 | 29039 | PB | Treatment 6 month later | uninfluenced | inhibited |
| GSE81622.gpl10558.test1 | 30506 | PBMC | LN vs Norm | activated | activated |
| GSE99967.gpl21970.test1 | 32677 | PB | LN vs Norm | uninfluenced | activated |

**GSE72747**: From patients with LN 6 months after initiation of conventional immunosuppressive therapy (*induction* during the first 3 months, followed by *maintenance*).

**GSE81622**: Whole-genome transcription analysis in PBMC of SLE patients with LN.

**GSE99967**: Whole blood RNA abundance profile of active SLE patients in the presence of LN.

**Supplement Table 5.** Comparison of laboratory parameters of systemic lupus erythematosus patients with and without lupus nephritis (LN).

|  | Active LN  (n=9) | Inactive LN  (n=9) |
| --- | --- | --- |
| **Age** | 17.11± 8.328 | 17.00± 4.243 |
| **Sex** | F:M=8:1 | F:M=7:2 |
| **SLEDAI** | 7.875±4.203 | 8.160±4.391 |
| **anti-dsDNA (IU/mL)** | 520.4±302.8**** | 306.0±336.6 |
| **C3 (mg/dL)** | 79.95±23.48 | 76.14±25.64 |
| **C4 (mg/dL)** | 12.53±5.854**** | 17.82±7.164 |
| **WBC (K/μL)** | 5.563±3.031** | 6.511±2.558 |
| **Hb (g/dL)** | 10.85±1.695 | 11.22±2.999 |
| **Platelet (K/μL)** | 291.3±94.75*** | 223.2±88.08 |
| **ESR (mm /hr)** | 42.87±31.57**** | 18.87±20.16 |
| **CRP (mg/dL)** | 0.3258±0.6081* | 0.1518±0.2919 |
| **serum Creatinine (mg/dL)** | 0.5429±0.1174 | 0.736±0.3966 |
| **eGFR (mL/min/1.73 m^2^)** | 82.3±21.73 | 80.52±49.82 |

*: *P*<0.05; **: *P*<0.01; ***: *P*<0.001; ****: *P*<0.0001.
